# The Departed

**Published:** 1872-05

**Authors:** 


### OBITUARY.

### The Departed.

The profession will receive the intellegence of the death of Dr. R. W. Varney with surprise and sadness. He was one of the most prominent young men in the profession, no one of his age stood higher; he has done much for the improvement and advancement of dental practice, by quiet, persevering, efficient work, rather than by any ostentatious display; all that he did had the stamp of superiority upon it: indeed it is claimed by some who knew him best that he had no superior if an equal in the world as an operator. He was possessed of great, natural ability, and added to this the most thorough education and training under the best masters; this with an indomitable determination to excell made him what he was. He was social, kind-hearted and generous to a high degree; and those who knew him best loved him most. In his departure the profession will perhaps never realize the extant of the loss; but though they have lost, he has gained.

